# Very-early-onset FPIES and its difficult management

**DOI:** 10.1186/2045-7022-1-S1-P118

**Published:** 2011-08-12

**Authors:** Daniele Ghiglioni, Oscar Mazzina, Elena Calcinai, Marco Albarini, Marco Trezzi, Alessandro Fiocchi

**Affiliations:** 1 Melloni Paediatria, Milan, Italy; 2 PLADA SpA, Milan, Italy

## 

A 6-month-old girl presented referred for FPIES of difficult management. Admitted for suspected sepsis at 15 days after receiving milk thickened with cream of rice, she was hospitalized for suspected anaphylaxis at 5 months soon after her first rice-containing meal. Correctly diagnosed with rice-induced FPIES, she was challenged with maize to which she reacted with vomiting, hypotonia and bloody diarrhoea. Referred to this institution for investigation of cereal tolerance, we suspected industrial maize food contamination with rice and requested from a manufacturer a whole wheat flour which was tolerated at challenge. The girl reacted strongly to two accidental contacts with rice. A special diet was initiated in collaboration with the manufacturers who used the same rice-free flour to make pasta to which she did not react. Introduction of solids was predicated on a series of challenges, including beef, carrot, potato, pork, chicken, zucchini and spinach. Of note, after 5 symptom-free months, the girl reacted to accidental ingestion of a fragment of a sweet left over from the day before.

This case report highlights how FPIES onset can be very early, though patients may respond to dietary management with cross-contaminant-free baby foods.

